# Neuroimaging Markers of Cerebral Small Vessel Disease on Hemorrhagic Transformation and Functional Outcome After Intravenous Thrombolysis in Patients With Acute Ischemic Stroke: A Systematic Review and Meta-Analysis

**DOI:** 10.3389/fnagi.2021.692942

**Published:** 2021-07-13

**Authors:** Yiqiao Wang, Xiaoting Yan, Jie Zhan, Peiming Zhang, Guangming Zhang, Shuqi Ge, Hao Wen, Lin Wang, Nenggui Xu, Liming Lu

**Affiliations:** ^1^South China Research Center for Acupuncture and Moxibustion, Medical College of Acu-Moxi and Rehabilitation, Guangzhou University of Chinese Medicine, Guangzhou, China; ^2^Postdoctoral Programme, The Second Affiliated Hospital of Guangzhou University of Chinese Medicine, Guangzhou, China; ^3^Department of Neurology, The Sun Yat-sen Memorial Hospital of Sun Yat-sen University, Guangzhou, China

**Keywords:** cerebral small vessel disease, acute ischemic stroke, intravenous thrombolysis, hemorrhagic transformation, neuroimaging markers

## Abstract

**Objective:** The aim of this study was to perform a systematic review and meta-analysis to assess whether cerebral small vessel disease (CSVD) on neuroimaging of patients with acute ischemic stroke (AIS) treated with intravenous thrombolysis (IVT) is associated with an increased risk of hemorrhagic transformation (HT), symptomatic intracranial hemorrhage (sICH), and poor functional outcome (PFO).

**Methods:** A thorough search of several databases was carried out to identify relevant studies up to December 2020. We included studies of patients with AIS and neuroimaging markers of CSVD treated with IVT. The primary outcome was HT, and the secondary outcomes were sICH and 3-month PFO. The quality of the studies involved was evaluated using the Newcastle–Ottawa Scale (NOS). The meta-analysis with the fixed effects model was performed.

**Results:** Twenty-four eligible studies (*n* = 9,419) were pooled in the meta-analysis. All included studies were regarded as high quality with the NOS scores of at least 6 points. The meta-analysis revealed associations between the presence of CSVD and HT, sICH, and the 3-month PFO after IVT. Compared with no CSVD, the presence of CSVD was associated with an increased risk of HT (OR: 1.81, 95% CI: 1.52–2.16), sICH (OR: 2.42, 95% CI: 1.76–3.33), and 3-month PFO (OR: 2.15, 95% CI: 1.89–2.44). For patients with AIS complicated with CSVD, compared with a CSVD score of 0–1, a CSVD score of 2–4 was associated with an increased risk of HT (OR: 3.10, 95% CI: 1.67–5.77), sICH (OR: 2.86, 95% CI: 1.26–6.49), and 3-month PFO (OR: 4.58, 95% CI: 2.97–7.06).

**Conclusion:** Patients with AIS complicated with neuroimaging markers of CSVD are at increased risk of HT and 3-month PFO after IVT. However, it is still necessary to clarify the exact role of CSVD in the occurrence, development, and prognosis of AIS.

**Systematic Review Registration:** www.ClinicalTrials.gov, identifier CRD4202123 3900.

## Introduction

Stroke is the second leading cause of death worldwide, leading to death in 5.5 million people and affecting 13.7 million each year. Ischemic stroke accounts for 70% of all patients with stroke. The 2016 Global Burden of Disease Study data that were published in 2019 suggest that one in four adults is reported to be at risk of having a stroke in their lifetime (GBD 2016 Stroke Collaborators, 2019; Lindsay et al., 2019).

Cerebral small vessel disease (CSVD) is a widespread cerebrovascular disease with specific neuroimaging characteristics (Chen et al., 2019). With the development of neuroimaging technology, the brain imaging of more patients with acute ischemic stroke (AIS) has detected the neuroimaging markers of CSVD such as cerebral microbleed (CMB), white matter hyperintensity (WMH), lacunar infarction (LI), and enlarged perivascular space (EPVS) (Curtze et al., 2016; Chen et al., 2019). CSVD accounts for 25% of cases of AIS, and it affects cognitive function, gait disturbance, swallowing, and other functions (Pantoni, 2010).

Studies have shown that CSVD may be a risk factor for intracranial hemorrhage, but none of these studies have a clear determinism (Emberson et al., 2014; Jickling et al., 2014; Pang et al., 2019). Hemorrhagic transformation (HT) occurs in 10–40% of patients with ischemic stroke and is a major complication of intravenous thrombolysis (IVT) (Terruso et al., 2009; Beslow et al., 2011; Jickling et al., 2014). HT can be divided into symptomatic and asymptomatic according to the deterioration of neurological function, both of which worsen the prognosis of stroke, especially in cognitive and neurological functions (Dzialowski et al., 2007; Park et al., 2012). Most patients with AIS complicated with CSVD have no obvious clinical symptoms at the initial stage, which are easy to be ignored by doctors. Therefore, for patients with AIS complicated with CSVD, HT, symptomatic intracranial hemorrhage (sICH), and poor functional outcome (PFO) after IVT have gradually attached the attention of medical researchers (Liu X.Y. et al., 2019). Different subtypes of CSVD may have different effects on HT and clinical prognosis after IVT in patients with AIS, and different subtypes of CSVD often coexist in the same patients with AIS.

Several recent studies have explored the relationship between these neuroimaging markers of CSVD and clinical outcomes after IVT in patients with AIS (Drelon et al., 2020; Liu X. et al., 2020). Previous meta-analyses have investigated the increased risk of CSVD for HT and PFO in patients with AIS (Charidimou et al., 2016; Tsivgoulis et al., 2016; Wang et al., 2017). However, these studies are mainly limited to a particular subtype of CSVD, and some patients receive endovascular treatment. Whether the existence of neuroimaging markers of CSVD affects the HT, sICH, and PFO of patients with AIS after IVT is still a controversial issue. Therefore, we performed a systematic review and meta-analysis to evaluate whether CSVD on neuroimaging of patients with AIS treated with IVT is associated with an increased risk of HT, sICH, and PFO.

## Methods

This systematic review and meta-analysis was performed in accordance with the Preferred Reporting Items for Systematic reviews and Meta-Analyses (PRISMA) guidelines.

### Search Strategy

We systematically searched the MEDLINE, Cochrane Library, Embase, CNKI, VIP, and WANFANG databases from inception to December 2020 to find relevant studies. The articles were not restricted based on the language of publication. The details of the search strategy are presented in Supplementary Appendix 1. Two authors (i.e., WYQ and YXT) scanned the titles and abstracts to find the articles that were most relevant to this study; then, the full texts of the relevant articles were examined, and the final decision on inclusion was made by consensus.

### Selection Criteria

#### Types of Studies

We included cohort studies (i.e., prospective and retrospective) in which patients with AIS or suspected AIS were treated with IVT. The relationship between neuroimaging markers of CSVD and clinical outcomes was assessed. All eligible trials were published in full text without language restrictions.

#### Types of Participants

We considered trials that included IVT-treated patients with AIS or patients who were treated with IVT for suspected ischemic stroke. The diagnosis of AIS meets the WHO diagnostic criteria (No authors listed, 1989), and participants were confirmed by CT or MRI. The diagnosis points of AIS are as follows: (1) acute onset, (2) focal neurological deficit, (3) responsible lesions appearing on imaging or symptoms lasting more than 24 h, and (4) excluding cerebral hemorrhage by brain CT/MRI. Trials involving patients treated with endovascular therapy were excluded. Cerebral imaging had to be performed for the visualization of CSVD. CMB, WMH, LI, and EPVS can all be detected on MRI (Chen et al., 2019). CMB is defined as a small, round, or oval hypointense lesion that can be shown on T2^∗^-weighted gradient recalled echo (GRE) and susceptibility-weighted imaging (SWI) (Wardlaw et al., 2013). WMH is an imaging description of white matter demyelination, it is hyperintense on T2-weighted image (T2WI) and fluid-attenuated inversion recovery (FLAIR) sequences on MRI, and it can also be shown on CT, but the range of lesions shown on CT may not be ideal (Wardlaw et al., 2013; van Leijsen et al., 2018). LI is a hyperintense area with the largest lesion diameter less than 20 mm on the axial plane of the FLAIR sequence (Wardlaw et al., 2013). Generally, the diameter of the perivascular space is less than 2 mm, the EPVS can extend to a diameter of 2–4 mm, and it can be detected on MRI (Chen et al., 2019). To expand the scope of the study, WMH detected on CT is also included (Charidimou et al., 2016).

#### Primary Outcome Assessments

The primary outcome for the systematic review was the occurrence of HT. There are many types of HT diagnostic criteria, such as the European Cooperative Acute Stroke Studies (ECASS) (Fiorelli et al., 1999; Larrue et al., 2001; Hacke et al., 2008) and the National Institute of Neurological Disorders and Stroke (NINDS) (No authors listed, 1997). The diagnosis points of ECASS are as follows: (1) hemorrhagic infarction (HI): there was a small spot-like hemorrhage along the edge of the infarct and sheet-like non-massive bleeding or multiple fused spot-like hemorrhages in the infarct area and (2) parenchymal hematoma (PH): hematoma, i.e., bleeding with slight or obvious space-occupying effect or bleeding away from the infarct. The main points of the diagnosis of NINDS are as follows: (1) HI: different low-density/high-density foci with punctate or blurred borders in the acute infarct can be tested on CT and (2) PH: typical homogeneous high-density lesions with clear boundaries, with or without cerebral edema or space-occupying effects, can be tested on CT.

#### Secondary Outcome Assessments

The secondary outcomes included the occurrence of sICH, and sICH was defined by the scores on the ECASS (Fiorelli et al., 1999; Larrue et al., 2001; Hacke et al., 2008), NINDS (No authors listed, 1997), and Safe Implementation of Thrombolysis in Stroke-Monitoring Study (SITS-MOST) (Rha et al., 2014). The 3-month PFO was defined as a Modified Rankin Scale (mRS) score > 2. The relationships among HT, sICH, 3-month PFO, and the total burden of CSVD (by using the Total Burden Rating Scale of CSVD) were also examined. The diagnosis points of ECASS are as follows: bleeding is seen on CT, and the increase in the National Institute of Health Stroke Scale (NIHSS) score is ≥ 4 points. The diagnosis points of NINDS are as follows: bleeding on CT, accompanied by neurological decline. The main points of diagnosis of SITS-MOST are as follows: the infarct area or remote area PH is seen on CT, the NIHSS score increased by 4 points or more compared with the minimum level of 24 h after admission, and bleeding caused death. The mRS can assess the complete independent living ability of patients with stroke, where a score of 0 means asymptomatic and the higher scores indicate a worse prognosis (i.e., a score of 6 indicates death). The concept of the total burden of CSVD was proposed by Staals et al. (2014), who developed a scale to quantitatively evaluate the cumulative effect of CSVD throughout the whole brain. The total score of the scale ranges from 0 to 4 points, and the higher the score, the more serious the CSVD.

### Data Extraction

We used a preset electronic collection form to extract the basic characteristics of the studies, such as study design, first author, year of publication, country, sample size, age, sex, imaging method, type of neuroimaging markers of CSVD (i.e., CMB, WMH, LI, and EPVS), the definition of HT and sICH, the total burden of CSVD, and clinical outcome. The number of patients with neuroimaging markers of CSVD and the number of outcome events in each study were extracted, and conversion was performed when studies were reported as a percentage. When a study reported the relationship between different subtypes of CSVD and outcomes, the data were extracted separately.

### Quality Assessment

Two reviewers independently assessed each study for quality using the Newcastle–Ottawa Scale (NOS) (Wells et al., 2013), which mainly contains three domains as follows: (1) selection, (2) comparability, and (3) outcome. The selection domain included the representativeness of the exposed cohort, selection of non-exposed cohort, ascertainment of exposure, and demonstration that the outcome of interest was not present at the beginning of the study. The comparability domain included the comparability of cohorts based on the design or analysis. Outcomes included the assessment, long enough follow-up for outcomes to occur, and adequacy of follow-up of cohorts. The maximum score of the NOS was 9 points, and a score ≥ 6 indicated a high-quality study. Disagreements in the studies were resolved by senior researchers. The details of the quality assessment can be found in Supplementary Appendix 2.

### Statistical Analyses

We used RevMan software version 5.3 and Stata MP software version 14.0 for all the statistical analyses. The meta-analysis was used to calculate the combined odds ratio (OR) and the corresponding 95% CI to quantify the strength of the association between the presence and severity of CSVD and HT, sICH, and 3-month PFO after IVT. The total burden of CSVD was divided into two groups, namely, 0–1 and 2–4 points. We tested for heterogeneity between trial results using the *I*^2^ statistic (Higgins et al., 2003) (when *P* ≤ 0.1 and *I*^2^ ≤ 25% indicated low heterogeneity, 25% < *I*^2^ ≤ 50% indicated moderate heterogeneity, and *I*^2^ > 50% indicated significant heterogeneity). The fixed effects model was used when heterogeneity between studies was not detected; otherwise, the random effects model was used.

To explore the factors associated with heterogeneity, the subgroup analysis according to different types of CSVD (i.e., CMB, WMH, LI, and EPVS) was performed to explore the impact of different types on the outcome. The sensitivity analysis was used to analyze whether the conclusion was stable, and we eliminated each study one by one to explore its influence on the conclusion. We planned to use funnel plots to visually observe the possibility of reporting bias and combined the Egger’s test to detect publication bias (Egger et al., 1997). Two-sided *P* ≤ 0.05 was considered statistically significant.

## Results

### Review of the Literature

**FIGURE 1:**
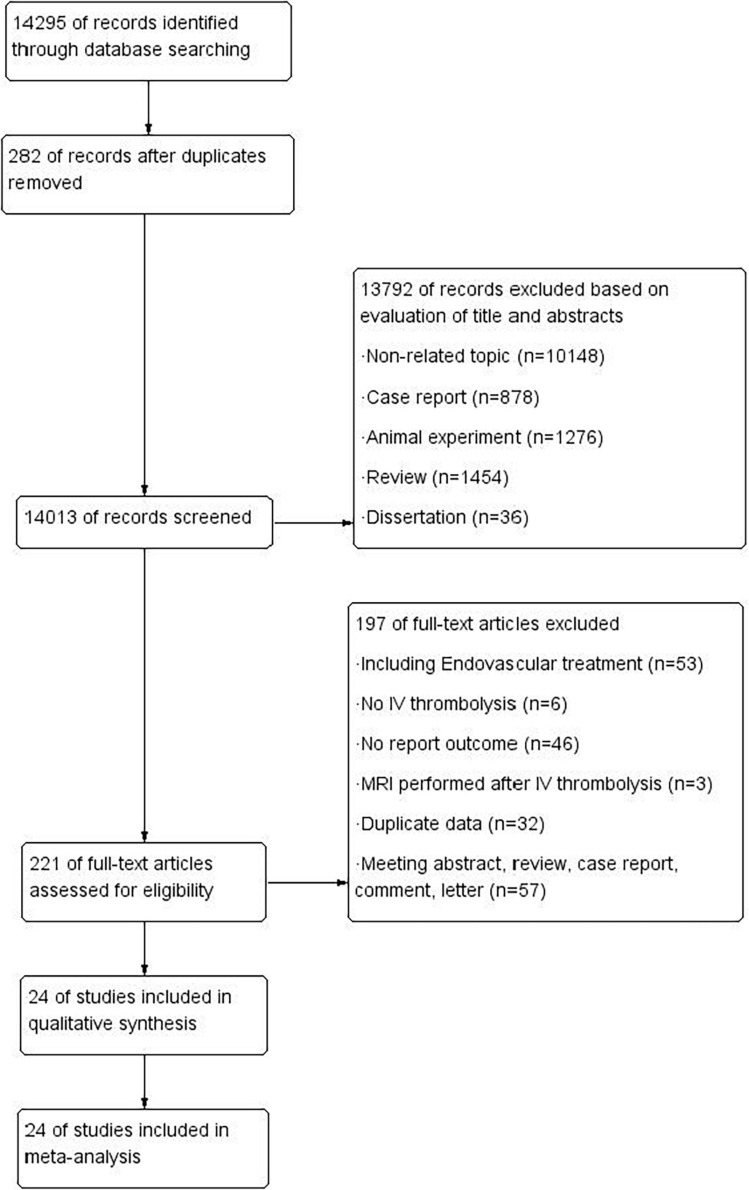
Flow diagram of the search process and study selection.

Our initial search yielded 14,295 potentially related studies. Of note, 282 studies were excluded because they were duplicates, 13,792 were excluded based on titles and abstracts, and 197 were excluded after a full-text reading revealed that the studies did not meet the inclusion criteria. Finally, for a total of 9,419 patients, 24 studies (Derex et al., 2004; Kakuda et al., 2005; Zheng et al., 2012; Moriya et al., 2013; Dannenberg et al., 2014; Yan et al., 2014, 2015a,b; Curtze et al., 2015a,b; Liu et al., 2017, 2018; Huang, 2017; Xue et al., 2017; Chacon-Portillo et al., 2018; Nagaraja et al., 2018; Dong and Xia, 2018; Zhang and Zhang, 2018; Liu L. et al., 2019; Liu et al., 2019a; Liu L. et al., 2019; Wen et al., 2019; Capuana et al., 2020; Zhuo et al., 2020) meeting the criteria were included. The selection process is shown in Figure 1. These studies were cohort studies published from the year 2004 to 2020 and included 6 prospective and 18 retrospective studies. Among them, 17 studies evaluated HT, 11 studies mentioned sICH, and 10 studies assessed 3-month PFO. Three of these studies reported separate associations with the total burden of CSVD. In the included studies, the neuroimaging markers of CSVD included cerebral CMB, WMH, and LI, and no studies related to EPVS were found. The characteristics of these studies are shown in Table 1.

**TABLE 1:** Characteristics of the studies in the meta-analysis.

**Author (year)**	**Period**	**Country**	**Study design**	**Sample**	**Mean/Median Age(y)**	**Gender (Female/male)**	**CVSD**	**Clinical outcomes**	**Imaging method**	**Time-HT/sICH**	**NOS**
Moriya et al. (2013)	2007.11–2011.12	Japan	Prospective	71	73 ± 10	21/50	CMB	HT(NINDS)	T2*-GRE(1.5T), FLAIR, DWI, T1WI, T2WI	Between days 4 and 7 after admission	6
Capuana et al. (2020)	NA	Italy	Retrospective	434	68.3 ± 13.5	170/264	CMB	HT(ECASS **II**) sICH(ECASS)	T2*-GRE(1.5T), T1WI, T2WI, FLAIR, DWI, PWI, 3d-TOF-MRA	Within 24 h after IVT	8
Liu et al. (2019b)	2016.08–2018.07	China	Prospective	218	65.79 ± 11.99	144/74	The total burden of CSVD, WMH	PFO	T2*-GRE (-), FLAIR, T2WI, T1WI, DWI, 3d-TOF-MRA	/	8
Wen et al. (2019)	2016.09–2019.01	China	Retrospective	326	Good outcome:65 ± 12 Poor outcome: 70 ± 12	117/209	WMH	PFO	T1WI, T2WI, FLAIR	/	8
Liu L. et al. (2019)	2017.01–2019.01	China	Retrospective	175	67 ± 11	62/113	The total burden of CSVD	PFO, HT (ECASSII), sICH (ECASSII)	DWI, FLAIR, SWI, MRA	Within 24 h after IVT	8
Liu X.Y. et al. (2019)	2016.08–2018.01	China	Retrospective	154	66.00 (59.00, 74.25) **(**Median;Interquartile range)	51/103	The total burden of CSVD, CMB, Li, CMB, LI	HT(ECASSII), sICH(NINDS)	T1WI, T2WI, FLAIR, DWI	Within 24 h after IVT	6
Chacon-Portillo et al. (2018)	2011.01–2015.12	America	Retrospective	292	62.8 ± 15.3	151/141	CMB	sICH (NINDS), HT(ECASSII)	T1WI, T2WI, DWI, SWI	Within 24 h after IVT	6
Nagaraja et al. (2018)	2009.01–2013.12	America	Retrospective	366	67 ± 15	168/198	CMB	HT (ECASSI)	T2*-GRE(1.5T), DWI, FLAIR, CT (HT)	At 18–36 h after IVT	6
Liu et al. (2018)	2013.06–2017.05	China	Retrospective	97	66.6 ± 9.1	29/68	WMH	PFO, HT(ECASSII)	DWL, FLAIR, MRA-, CT (HT)	Within 24 h after IVT	8
Liu et al. (2017)	2014.01–2017.03	China	Retrospective	78	WMH: 73.7 ± 6.7 No WMH:61.3±10.6	24/54	WMH, LI	PFO, HT(ECASSII), sICH (ECASSII)	T1WI, FLAIR, DWI, MRA	Within 24 h after IVT	8
Yan et al. (2015a)	2009.06–2015.06	China	Retrospective	449	66.8 ± 12.9 SICH: 73 (64, 79)	151/298	CMB	PFO, HT(ECASSII), sICH(ECASSII)	SWI	Within 24 h after IVT	7
Curtze et al. (2015a)	2001.12–2014.02	UK	Retrospective	2481	No SICH:69 (60, 77) (Median; Interquartile range)	1,074/1,407	WMH	sICH(ECASSII)	CT	At 24 h post IVT	6
Dannenberg et al. (2014)	2008.01–2013.08	Germany	Prospective	326	76 (68, 84) (Median; Interquartile range)	167/159	CMB	sICH(ECASSIII)	T2*-GRE(3T), DWI	Within 36 h after IVT	8
Yan et al. (2014)	2009.06–2013.05	China	Retrospective	225	66.29 ± 13.01	73/152	CMB	HT(ECASSII)	FLAIR, DWI, PWI, SWI, MRA	Within 24 h after IVT	8
Zheng et al. (2012)	2006.07–2011.10	China	Retrospective	175	68 ± 10	70/105	WMH	HT(ECASSII)	CT, T2WI, FLAIR	Within 24 h after IVT	7
Kakuda et al. (2005)	2001.04–2005.01	America, Canada, Belgium	Prospective	70	71 ± 29	39/31	CMB	HT(ECASS) sICH(ECASS)	GRE(1.5T), DWI, PWI, MRA, T1WI	At 3–6 h after IVT and at day 30	8
Derex et al. (2004)	2001.05–2002.08	France	Retrospective	44	63.2 ± 14.1	21/23	CMB	HT(NINDS), sICH(NINDS)	T2*-GRE(1.5T), CT(HT, SICH)	At day 7	6
Yan et al. (2015a)	2009.06–2014.02	China	Retrospective	333	66.15 ± 13.02	110/223	CMB	HT(ECASSII), PFO	GRE(3.0T), DWI, SWI	At 24 hours post IVT	6
Dong and Xia (2018)	2015.01–2016.12	China	Prospective	56	69.26 ± 2.25	25/31	CMB	HT(ECASS)	T1WI, T2WI, T2FLAIR, DWI, MRA, SWI	At 24 hours post IVT	6
Zhang and Zhang (2018)	2015.07–2016.07	China	Retrospective	206	CMB: 63.2 ± 9.5 NO CMB: 61.5 ± 9.0	83/123	CMB	sICH(SITS-MOST), PFO	T2*-GRE(-), T1WI, T2WI, DWI	NA	6
Huang (2017)	2010.01–2016.01	China	Retrospective	100	18–80	42/58	CMB	HT(ECASSI)	SWI, CT(HT)	At 24 hours post IVT	6
Zhuo et al. (2020)	2012.03–2018.01	China	Retrospective	178	62.3 ± 10.5	53/125	CMB, LI	PFO	T1WI, T2WI, DWI, FLAIR, SWI	/	8
Xue et al. (2017)	2012.01–2015.06	China	Prospective	80	56 ± 12	22/58	CMB	HT(ECASSII)	DWI, MRA, SWI	At 24 ± 12 hours post IVT	6
Curtze et al. (2015b)	2001.12–2014.02	UK	Retrospective	2485	78 (72–83)	1076/1409	WMH	PFO	CT	/	6

CMB, cerebral microbleed; WMH, white matter hyperintensity; LI, lacunar infarction; The total burden of CSVD, the total burden of cerebral small vessel disease; NA not available; HT, hemorrhagic transformation; sICH, symptomatic intracranial hemorrhage; PFO, poor functional outcome; ECASS, European Cooperative Acute Stroke Studies; SITS-MOST, safe implementation of thrombolysis in stroke-monitoring study; NINDS, the National Institute of Neurological Disorders and Stroke; T1WI, T1 Weighted Image; T2WI, T2 Weighted Image; FLAIR, Fluid attenuated inversion recovery; SWI, susceptibility-weighted imaging; T2*-GRE, T2*-weighted gradient-recalled echo; DWI, Diffusion-Weighted Imaging; PWI, Perfusion-Weighted Imaging; CT, Computed Tomography; MRA, Magnetic Resonance Angiography; 3d-TOF-MRA, 3D time-of-flight Magnetic Resonance Angiography; CT (HT, sICH), CT was used to detected HT, sICH.

### Neuroimaging Markers of CSVD and HT

**FIGURE 2:**
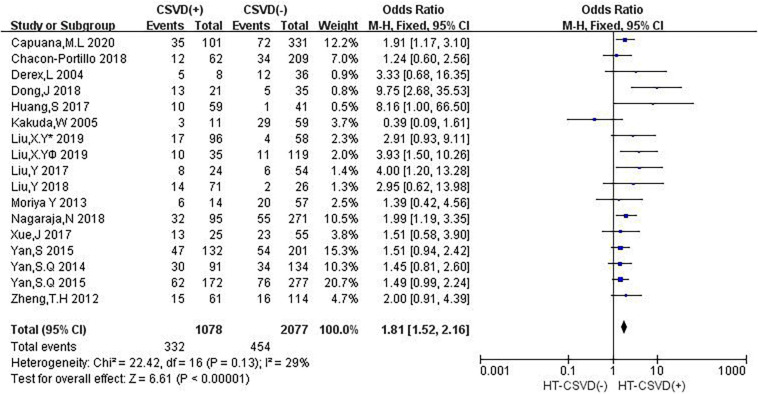
Forest plot showing the Impact of CSVD on HT (Liu, XY^*v*^ 2019 and Liu, XY^®^ 2019 are from Liu et al. (2019a); Liu, XY^¥^ 2019 represents the data of LI in this study, and Liu, Y^∗^ 2019 represents the data of CMB in this study).

Sixteen studies evaluated the relationship between neuroimaging markers of CSVD and HT. The pooled overall rate of HT after IVT was 24.9% in the entire population. The pooled rate was 30.8% of patients in the CSVD presence group vs. 21.9% of patients in the group without CSVD presence. The total number of participants was 3,155 patients. Compared with no CSVD, the presence of CSVD was associated with an increased risk of HT (OR: 1.81, 95% CI: 1.52–2.16) (Figure 2). The heterogeneity between studies was not significant (*I*^2^ = 29%, *P* = 0.13). The risk of HT after IVT was higher in patients with CSVD than in patients without CSVD on neuroimaging.

**FIGURE 3:**
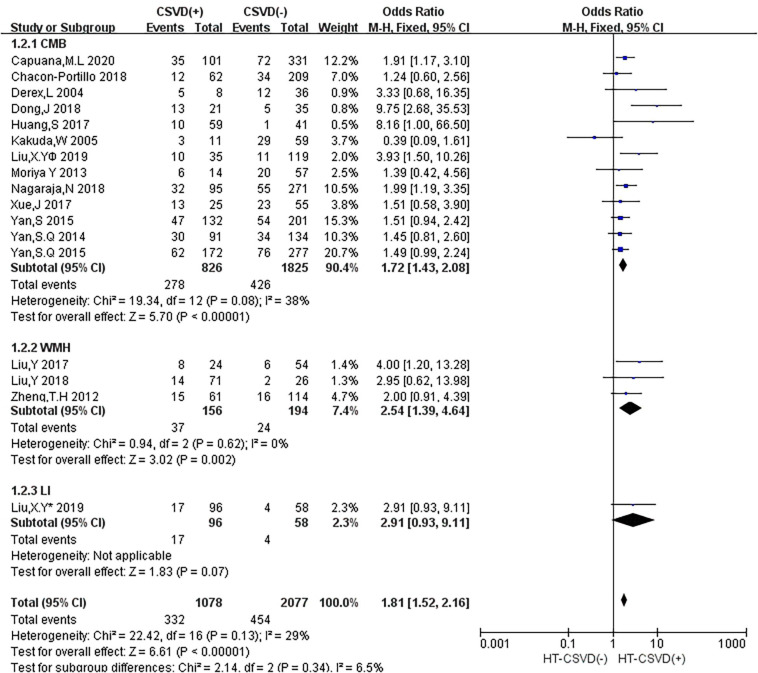
Forest plot of subgroup analysis showing the impact of subtypes of CSVD on HT.

In subgroup analyses, the influence of CSVD on HT showed a few changes when the studies were stratified according to the type of CSVD (Figure 3). Twelve studies (50%) reported the relationship between CMB and HT, three studies (12.5%) evaluated the association between WMH and HT, and one study (4.2%) mentioned the relationship between CMB and HT as well as LI and HT. Compared with no CMB, the presence of CMB was associated with an increased risk of HT (OR: 1.72, 95% CI: 1.43–2.08), and moderate heterogeneity was observed (*I*^2^ = 38%, *P* = 0.08). Compared with no WMH, the presence of WMH was associated with an increased risk of HT (OR: 2.54, 95% CI: 1.39–4.64), and there was no heterogeneity (*I*^2^ = 0, *P* = 0.62). An estimate specific for LI and HT was provided in only one study (OR: 2.91, 95% CI: 0.93–9.11).

**FIGURE 4:**
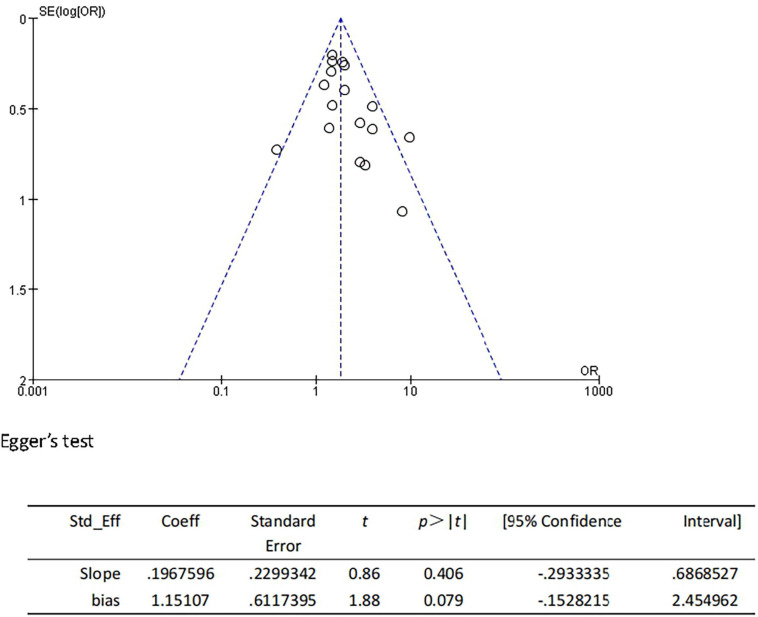
Funnel plot and Egger’s test of studies evaluating the association between CSVD and HT.

We performed the sensitivity analyses to explore whether the change in the inclusion criteria of studies influenced the robustness of the combined results. We recalculated the pooled OR by excluding each individual study in turn. The range of the combined ORs was from 1.75 (95% CI: 1.46–2.09) to 1.89 (95% CI: 1.56–2.30) when the studies of Dong and Xia (2018) and Yan et al. (2015a) were excluded. The results showed that no individual study significantly affected the pooled effect size. Furthermore, the funnel plots (Figure 4) and the Egger’s test (*t* = 1.88, *P* = 0.079) indicated no publication bias.

### Neuroimaging Markers of CSVD and sICH

**FIGURE 5:**
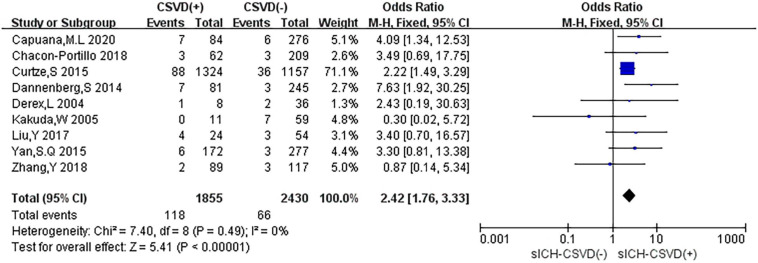
Forest plot showing the impact of CSVD on sICH.

Nine studies reported the relationship between neuroimaging markers of CSVD and sICH. The pooled overall rate of sICH after IVT was 4.3% in the entire population. The pooled rate was 6.4% of patients in the CSVD presence group vs. 2.7% of patients in the group without CSVD presence. The total number of participants was 4,285 patients. Compared with no CSVD, the presence of CSVD was associated with an increased risk of sICH (OR: 2.42, 95% CI: 1.76–3.33) (Figure 5). The heterogeneity was not detected (*I*^2^ = 0%, *P* = 0.49). The risk of sICH after IVT was found to be higher in patients with evidence of CSVD than in patients without CSVD on neuroimaging.

**FIGURE 6:**
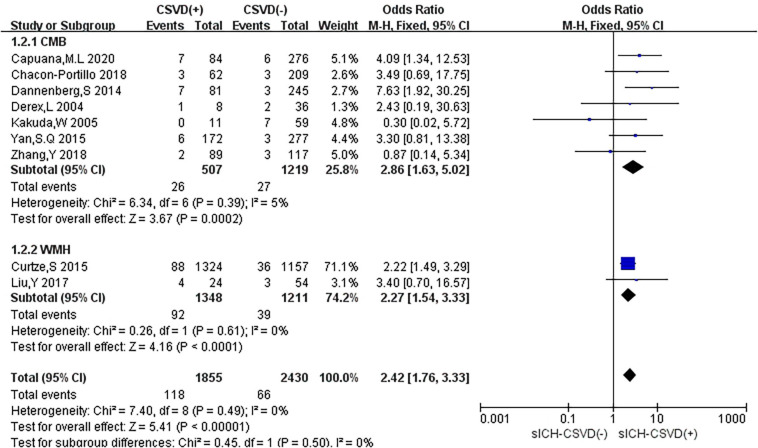
Forest plot of subgroup analysis showing the impact of subtypes of CSVD on sICH.

In subgroup analyses, different types of CSVD had slightly different effects on sICH (Figure 6). Seven studies (29.2%) reported the relationship between CMB and sICH, only two studies (8.3%) evaluated the association between WMH and sICH, and no study mentioned the correlation between LI and sICH. Compared with no CMB, the presence of CMB was associated with an increased risk of HT (OR: 2.86, 95% CI: 1.63–5.02), and low heterogeneity was observed (*I*^2^ = 5%, *P* = 0.39). Compared with no WMH, the presence of WMH was associated with an increased risk of sICH (OR: 2.27, 95% CI: 1.54–3.33), and there was no heterogeneity (*I*^2^ = 0%, *P* = 0.61).

**FIGURE 7:**
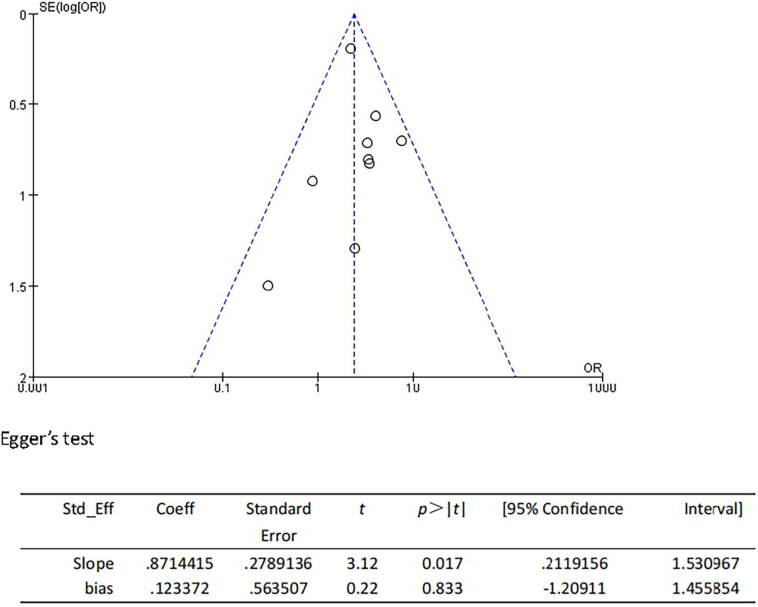
Funnel plot and Egger’s test of studies evaluating the association between CSVD and sICH.

We performed the sensitivity analyses to explore whether the outcome was stable. We recalculated the pooled OR by excluding each individual study in turn. The range of the combined OR was from 2.28 (95% CI: 1.64–3.16) to 2.92 (95% CI: 1.72–4.96) when the studies of Dannenberg et al. (2014) and Curtze et al. (2015a) were excluded. The results suggested that no individual study significantly affected the pooled effect size. The funnel plots (Figure 7) and the Egger’s test (*t* = 0.22, *P* = 0.833) indicated no evidence of publication bias.

### Neuroimaging Markers of CSVD and 3-Month PFO

**FIGURE 8:**
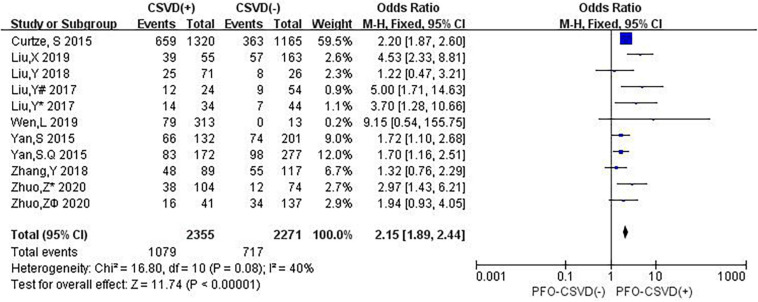
Forest plot showing the impact of CSVD on 3-month PFO. (Liu, Y^#^ 2017 and Liu, Y* 2017 are from Liu, Y 2017 (Liu et al., 2017); Liu, Y^#^ 2017 represents the data of WMH in this study, and Liu, Y* 2017 represents the data of LI in this study; both Zhuo Z* 2020 and Zhuo Z^ϕ^ 2020 are from Zhuo et al. (2020); Zhuo Z* 2020 represents the data of LI in this study, and Zhuo Z^ϕ^ 2020 represents the data of CMB in this study).

Nine studies investigated the relation between neuroimaging markers of CSVD and PFO. The pooled overall rate of 3-month PFO after IVT was 38.8% in the entire population. The pooled rate was 45.8% of patients in the CSVD presence group vs. 31.6% of patients in the group without CSVD presence. The total number of participants was 4,626, and compared with no CSVD, the presence of CSVD was associated with an increased risk of 3-month PFO (OR: 2.15, 95% CI: 1.89–2.44) (Figure 8). The heterogeneity between studies was moderate (*I*^2^ = 40%, *P* = 0.08). The results showed that patients with evidence of CSVD were at higher risk for PFO after IVT than patients without neuroimaging evidence of CSVD.

**FIGURE 9:**
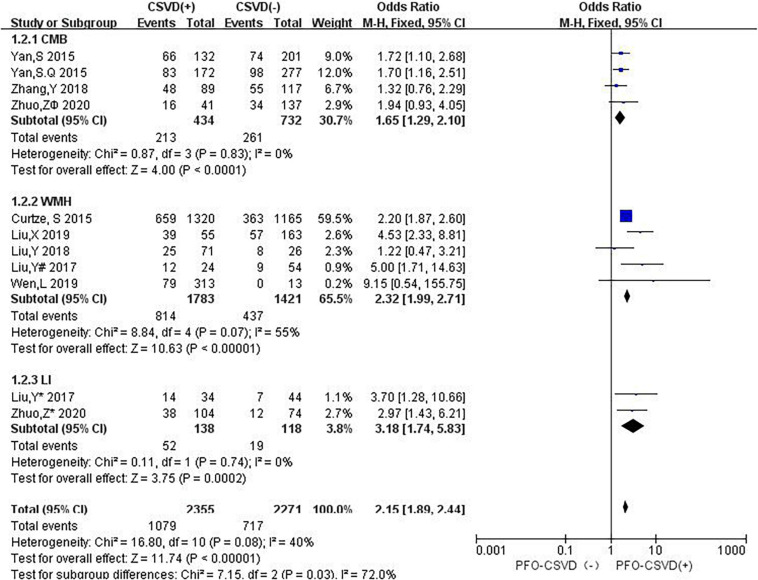
Forest plot of subgroup analysis showing the impact of subtypes of CSVD on 3-month PFO.

In subgroup analyses, the influence of CSVD on PFO showed some changes when studies were stratified according to the type of CSVD (Figure 9). Three studies (12.5%) reported the relationship between CMB and PFO, four studies (16.7%) evaluated the association between WMH and PFO, one study (4.2%) mentioned the relationship between CMB and PFO as well as LI and PFO, and one study (4.2%) reported the association between WMH and PFO as well as between LI and PFO. Compared with no CMB, the presence of CMB was associated with an increased risk of 3-month PFO (OR: 1.65, 95% CI: 1.29–2.10), and no heterogeneity was observed (*I*^2^ = 0%, *P* = 0.83). Compared with no WMH, the presence of WMH was associated with an increased risk of 3-month PFO (OR: 2.32, 95% CI: 1.99–2.71), and there was significant heterogeneity (*I*^2^ = 55%, *P* = 0.07). Compared with no LI, the presence of LI was associated with an increased risk of 3-month PFO (OR: 3.18, 95% CI: 1.74–5.83). There was no evidence of substantial heterogeneity among studies (*I*^2^ = 0%, *P* = 0.74).

**FIGURE 10:**
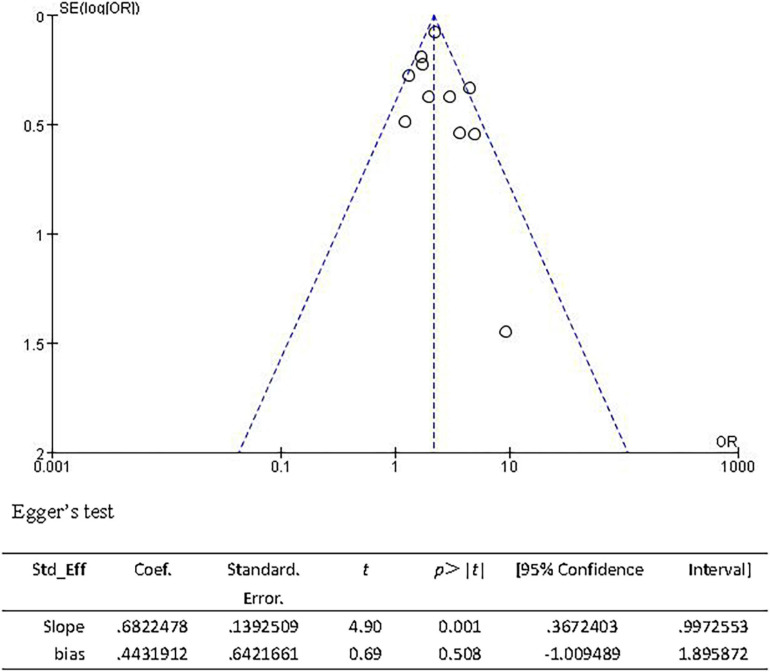
Funnel plot and Egger’s test of studies evaluating the association between CSVD and 3-month PFO.

The sensitivity analysis was carried out to explore the robustness of our analysis, and we recalculated the pooled OR by excluding each individual study in turn. The range of the combined ORs was from 2.06 (95% CI: 1.69–2.52) to 2.21 (95% CI: 1.93–2.52) when the studies of Curtze et al. (2015b) and Yan et al. (2015a) were excluded. The results showed that no individual study significantly affected the pooled effect size. The funnel plots (Figure 10) and the Egger’s test (*t* = 0.69, *P* = 0.508) showed no evidence of publication bias.

### The Total Burden of CSVD

**FIGURE 11:**
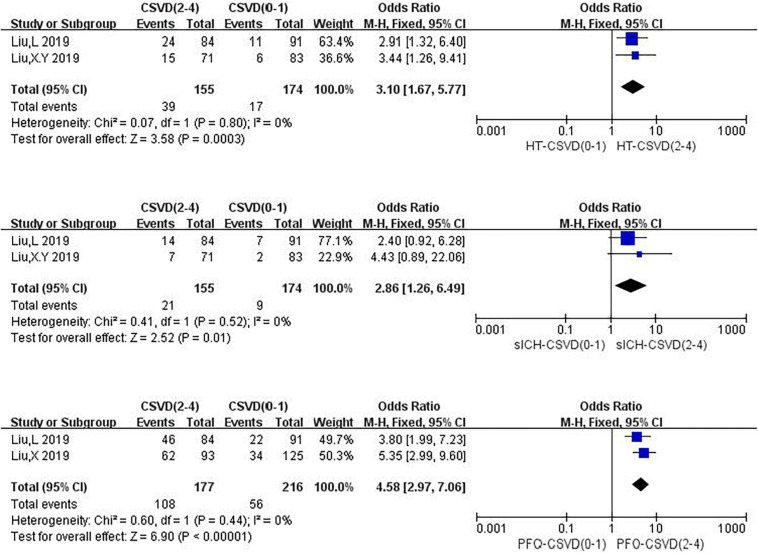
Forest plot showing the impact of CSVD score on HT, sICH and 3-months PFO.

Three studies were related to the total burden of CSVD, among which one explored the relationship between the total burden of CSVD and HT, sICH, and PFO (Figure 11). One study (4.2%) reported the relationship between the total burden of CSVD and HT as well as sICH, and one study (4.2%) investigated the association between the total burden of CSVD and PFO. We calculated pooled ORs of HT, sICH, and PFO for 2–4 CSVD scores vs. 0–1 CSVD scores. Compared with a CSVD score of 0–1, a CSVD score of 2–4 was associated with an increased risk of HT (OR: 3.10, 95% CI: 1.67–5.77), and no heterogeneity could be found (*I*^2^ = 0%, *P* = 0.80). Compared with a CSVD score of 0–1, a CSVD score of 2–4 was associated with an increased risk of sICH (OR: 2.86, 95% CI: 1.26–6.49), and there was no heterogeneity (*I*^2^ = 0%, *P* = 0.52). Compared with a CSVD score of 0–1, a CSVD score of 2–4 was associated with an increased risk of 3-month PFO (OR: 4.58, 95% CI: 2.97–7.06), and there was no evidence of substantial heterogeneity among studies (*I*^2^ = 0%, *P* = 0.44).

## Discussion

By using the systematic review and meta-analysis of these studies on the evidence of neuroimaging markers of CSVD, we observed that neuroimaging markers of CSVD (i.e., CMB, WMH, LI, and EPVS) represented an important indicator of a higher risk of HT, sICH, and 3-month PFO after IVT in patients with AIS. In addition, we found that a CSVD score of 2–4 (i.e., the Total Burden Rating Scale of CSVD) was a potential risk indicator for incidents. Our meta-analysis confirmed the association between the presence of neuroimaging markers of CSVD and the risk of HT and PFO after IVT. At present, there are many reports on the relationship between various subtypes of CSVD and HT and clinical prognosis after IVT in patients with AIS, but no definite conclusion has been reached. To the best of our knowledge, our study is the first to systematically evaluate the risk of HT and clinical prognosis in patients with AIS combined with neuroimaging markers of CSVD after IVT.

Cerebral small vessel disease is a widespread cerebrovascular disease that mainly manifests in CMB, WMH, LI, and EPVS on imaging. It mainly affects the perforation of small arteries, capillaries, and venules. The increased risk of HT and PFO in patients with AIS with CSVD after IVT may be related to the following mechanisms: First, the changes in blood pressure variability may have an effect on the increased risk. Studies have shown that increased blood pressure variability is closely related to the progression of CSVD, and increased blood pressure variability means increased blood pressure fluctuations, which increases the difficulty of maintaining the steady state of blood supply to the brain. The occurrence of hypoperfusion is more frequent (de Heus et al., 2020), which may be related to the occurrence of HT. Second, the destruction of the permeability of the blood–brain barrier, neuroimaging, and cerebrospinal fluid analysis indicate that leakage of the blood–brain barrier is a common feature of CSVD (Farrall and Wardlaw, 2009). When the permeability of the blood–brain barrier is broken, serum proteins and toxic effects will penetrate brain tissue, which may explain the complicated brain edema and poor functional prognosis in patients with AIS after IVT. Finally, the decrease in cerebral blood flow and vascular reactivity, the decrease in cerebral blood flow, and the decrease in vascular reactivity are related to CMB, WMH, LI, and EPVS (Gregg et al., 2015; Blair et al., 2016; Staszewski et al., 2019; Wang et al., 2020; Zhang et al., 2020). A previous meta-analysis of the cross-sectional studies revealed that patients with WMH had decreased cerebral blood flow (Shi et al., 2016), which may explain the PFO of patients with AIS after IVT. However, It is worth noting that IVT is still the standard treatment for patients with AIS within 4.5 h of onset (Powers et al., 2019). Although there is a 2–7% risk of sICH after IVT treatment, the risk of disability or death is absolutely reduced by 10% at 3 months (Turc et al., 2014). There is currently no research to prove that CSVD is an absolute contraindication for IVT in the treatment of AIS, several studies have shown that LI will benefit from IVT (Eggers et al., 2017; Matusevicius et al., 2019). Therefore, we cannot arbitrarily regard the neuroimaging markers of CSVD as an absolute contraindication for IVT based on the results obtained in our study.

Different subtypes of CSVD may have different effects on HT and the clinical prognosis of patients with AIS after IVT. Several meta-analyses have studied the prognosis of patients with CMB and WMH. Our results are consistent with a recent meta-analysis including 11 studies, which reported that WMH significantly increased the incidence of sICH after IVT and showed that the combined incidence of WMH and sICH was 1.55, and the OR for WMH and 3- to 6-month PFO was 2.02 (Charidimou et al., 2016). However, a previous updated meta-analysis on a similar topic was performed by Wang et al. (2017) including 11 studies with 2,702 patients and showed that CMB presence was not significantly associated with an increased risk of sICH. Compared with the previous meta-analysis, our results did not find such a connection. There are three possible reasons for this difference in the results. First, our study has a large sample size, including 24 articles (9,419 patients), and strict inclusion and exclusion criteria, while the earlier study only included 11 articles (2,702 patients). Second, in our study, the included patients with neuroimaging markers of CSVD could be tested by a variety of imaging methods, such as T1-weighted image (T1WI), T2WI, diffusion-weighted imaging (DWI), SWI, T2^∗^-GRE, while in a previous study (Wang et al., 2017), only SWI and T2^∗^-GRE were included in the imaging methods. Third, our results had low heterogeneity compared with previous studies (Charidimou et al., 2016; Wang et al., 2017).

Our study has the following strengthens. First, our study is the earliest to systematically evaluate the risk of HT and PFO in patients with AIS combined with the neuroimaging markers of CSVD after IVT. Second, we applied rigorous methodological standards, performed a systematic search, included a large sample size, and conducted an in-depth analysis of different subgroups. Our study also has some limitations as follows. First, there are differences in neuroimaging methods, both CT and MRI, which may affect the diagnosis of CSVD. Although the range of lesions shown on CT may not be ideal to expand the sample size, CT is also used to detect WMH. This may have a slight impact on the results of our study. However, we found that in previous studies, the application of CT to detect WMH was also be used for research (Charidimou et al., 2016). Second, the IVT protocol is different, and the baseline characteristics of the patient, such as previous use of anticoagulant drugs and repeated strokes, will affect the outcome. Third, the definitions of HT and sICH are different in each study. Although all we included are defined by standard criteria, they may still affect the results. However, in previous studies, the different definitions of HT and sICH, respectively, were combined for analysis (Charidimou et al., 2016; Wang et al., 2017). Fourth, in our study, in the subgroup analysis of CSVD and 3-month PFO, the presence of WMH was associated with the increased risk of 3-month PFO, and there was a significant heterogeneity. We found that the heterogeneity was observed from the study of Liu L. et al. (2019) probably due to the inclusion of participants, they excluded patients who had a premorbid mRS score > 2. Finally, there were no studies with EPVS, and only three studies mentioned the overall burden of CSVD.

## Conclusion

Our results indicate that patients with AIS complicated with the neuroimaging markers of CSVD are at an increased risk of HT and 3-month PFO after IVT. Our study strengthened the correlation between the neuroimaging markers of CSVD and the adverse outcome of AIS after IVT. However, it is undeniable that these patients can still benefit from IVT in some aspects. Therefore, it cannot be simply considered that the neuroimaging markers of CSVD are a contraindication for IVT in patients with AIS. It is still necessary to clarify the exact role of CSVD in the occurrence, development, and prognosis of AIS.

## Data Availability Statement

The original contributions presented in the study are included in the article/Supplementary Material, further inquiries can be directed to the corresponding author/s.

## Author Contributions

YW and LL were responsible for conception and design of this systematic review. YW drafted the manuscript. LL and NX revised the manuscript. YW, JZ, and SG designed the search strategies. YW, XY, and PZ conducted the electronic search. YW and XY extracted the data. YW and GZ assessed the risk of bias. YW, HW, and LW analyzed and interpreted the data. LL arbitrated any disagreements in the process of systematic review. All authors approved this manuscript.

## Conflict of Interest

The authors declare that the research was conducted in the absence of any commercial or financial relationships that could be construed as a potential conflict of interest.

## References

[B1] Beslow L. A. Smith S. E. Vossough A. Licht D. J. Kasner S. E. Favilla C. G. (2011). Hemorrhagic transformation of childhood arterial ischemic stroke. *Stroke* 42 941–946. 10.1161/strokeaha.110.604199 21350202 PMC3066279

[B2] Blair G. W. Doubal F. N. Thrippleton M. J. Marshall I. Wardlaw J. M. (2016). Magnetic resonance imaging for assessment of cerebrovascular reactivity in cerebral small vessel disease: a systematic review. *J. Cereb. Blood Flow Metab.* 36 833–841. 10.1177/0271678x16631756 26884471 PMC4853842

[B3] Capuana M. L. Lorenzano S. Caselli M. C. Paciaroni M. Toni D. (2020). Hemorrhagic risk after intravenous thrombolysis for ischemic stroke in patients with cerebral microbleeds and white matter disease. *Neurol. Sci.* 42 1969–1976. 10.1007/s10072-020-04720-y 32990857 PMC8043883

[B4] Chacon-Portillo M. A. Llinas R. H. Marsh E. B. (2018). Cerebral microbleeds shouldn’t dictate treatment of acute stroke: a retrospective cohort study evaluating risk of intracerebral hemorrhage. *BMC Neurol.* 18:33. 10.1186/s12883-018-1029-0 PMC5870091 29587638

[B5] Charidimou A. Pasi M. Fiorelli M. Shams S. von Kummer R. Pantoni L. (2016). Leukoaraiosis, cerebral hemorrhage, and outcome after intravenous thrombolysis for acute ischemic stroke: a meta-analysis (v1). *Stroke* 47 2364–2372. 10.1161/strokeaha.116.014096 27491738 PMC4995119

[B6] Chen X. Wang J. Shan Y. Cai W. Liu S. Hu M. (2019). Cerebral small vessel disease: neuroimaging markers and clinical implication. *J. Neurol.* 266 2347–2362. 10.1007/s00415-018-9077-3 30291424

[B7] Curtze S. Haapaniemi E. Melkas S. Mustanoja S. Putaala J. Sairanen T. (2015a). White matter lesions double the risk of post-thrombolytic intracerebral hemorrhage. *Stroke* 46 2149–2155. 10.1161/strokeaha.115.009318 26111888

[B8] Curtze S. Melkas S. Sibolt G. Haapaniemi E. Mustanoja S. Putaala J. (2015b). Cerebral computed tomography-graded white matter lesions are associated with worse outcome after thrombolysis in patients with stroke. *Stroke* 46 1554–1560. 10.1161/strokeaha.115.008941 25899244

[B9] Curtze S. Putaala J. Sibolt G. Melkas S. Mustanoja S. Haapaniemi E. (2016). Cerebral white matter lesions and post-thrombolytic remote parenchymal hemorrhage. *Ann. Neurol.* 80 593–599. 10.1002/ana.24760 27531598

[B10] Dannenberg S. Scheitz J. F. Rozanski M. Erdur H. Brunecker P. Werring D. J. (2014). Number of cerebral microbleeds and risk of intracerebral hemorrhage after intravenous thrombolysis. *Stroke* 45 2900–2905. 10.1161/strokeaha.114.006448 25116882

[B11] de Heus R. Reumers S. van der Have A. Tumelaire M. Tully P. J. Claassen J. A. H. R. (2020). Day-to-Day home blood pressure variability is associated with cerebral small vessel disease burden in a memory clinic population. *J. Alzheimers Dis.* 74 463–472. 10.3233/jad-191134 32039855 PMC7175928

[B12] Derex L. Nighoghossian N. Hermier M. Adeleine P. Philippeau F. Honnorat J. (2004). Thrombolysis for ischemic stroke in patients with old microbleeds on pretreatment MRI. *Cerebrovasc. Dis.* 17 238–241. 10.1159/000076123 14718753

[B13] Dong J. Xia Y. J. (2018). The application value of magnetic resonance SWI in the diagnosis and treatment of acute cerebral infraction. *J. Med. Imaging* 28 1423–1426.

[B14] Drelon A. Kuchcinski G. Caparros F. Dequatre-Ponchelle N. Moulin S. Cordonnier C. (2020). Remote brain hemorrhage after IV thrombolysis: role of preexisting lesions. *Neurology* 94 e961–e967. 31882531 10.1212/WNL.0000000000008874

[B15] Dzialowski I. Pexman J. H. Barber P. A. Demchuk A. M. Buchan A. M. Hill M. D. (2007). Asymptomatic hemorrhage after thrombolysis may not be benign: prognosis by hemorrhage type in the Canadian alteplase for stroke effectiveness study registry. *Stroke* 38 75–79. 10.1161/01.str.0000251644.76546.62 17122437

[B16] Egger M. Davey S. G. Schneider M. Minder C. (1997). Bias in meta-analysis detected by a simple, graphical test. *BMJ* 315 629–634. 10.1136/bmj.315.7109.629 9310563 PMC2127453

[B17] Eggers C. Bocksrucker C. Seyfang L. (2017). The efficacy of thrombolysis in lacunar stroke – evidence from the Austrian Stroke Unit Registry. *Eur. J. Neurol.* 24 780–787. 10.1111/ene.13288 28449276

[B18] Emberson J. Lees K. R. Lyden P. Blackwell L. Albers G. Bluhmki E. (2014). Effect of treatment delay, age, and stroke severity on the effects of intravenous thrombolysis with alteplase for acute ischaemic stroke: a meta-analysis of individual patient data from randomised trials. *Lancet* 384 1929–1935. 10.1016/s0140-6736(14)60584-5 25106063 PMC4441266

[B19] Farrall A. J. Wardlaw J. M. (2009). Blood-brain barrier: ageing and microvascular disease–systematic review and meta-analysis. *Neurobiol. Aging* 30 337–352. 10.1016/j.neurobiolaging.2007.07.015 17869382

[B20] Fiorelli M. Bastianello S. von Kummer R. del Zoppo G. J. Larrue V. Lesaffre E. (1999). Hemorrhagic transformation within 36 hours of a cerebral infarct: relationships with early clinical deterioration and 3-month outcome in the European Cooperative Acute Stroke Study I (ECASS I) cohort. *Stroke* 30 2280–2284. 10.1161/01.str.30.11.2280 10548658

[B21] GBD 2016 Stroke Collaborators (2019). Global, regional, and national burden of stroke, 1990-2016: a systematic analysis for the Global Burden of Disease Study 2016. *Lancet Neurol.* 18 439–458. 30871944 10.1016/S1474-4422(19)30034-1 PMC6494974

[B22] Gregg N. M. Kim A. E. Gurol M. E. Lopez O. L. Aizenstein H. J. Price J. C. (2015). Incidental cerebral microbleeds and cerebral blood flow in elderly individuals. *JAMA Neurol.* 72 1021–1028. 10.1001/jamaneurol.2015.1359 26167811 PMC4724412

[B23] Hacke W. Kaste M. Bluhmki E. Brozman M. Dávalos A. Guidetti D. (2008). Thrombolysis with alteplase 3 to 4.5 hours after acute ischemic stroke. *N. Engl. J. Med.* 359 1317–1329. 18815396 10.1056/NEJMoa0804656

[B24] Higgins J. P. Thompson S. G. Deeks J. J. Altman D. G. (2003). Measuring inconsistency in meta-analyses. *BMJ* 327 557–560. 10.1136/bmj.327.7414.557 12958120 PMC192859

[B25] Huang S. S. (2017). Cerebral microhemorrhage foci are associated with hemorrhage transformation and prognosis of patients with acute cerebral infarction after intravenous thrombolysis. *Med. J. Commun.* 31 63–65.

[B26] Jickling G. C. Liu D. Stamova B. Ander B. P. Zhan X. Lu A. (2014). Hemorrhagic transformation after ischemic stroke in animals and humans. *J. Cereb. Blood Flow Metab.* 34 185–199. 10.1038/jcbfm.2013.203 24281743 PMC3915212

[B27] Kakuda W. Thijs V. N. Lansberg M. G. Bammer R. Wechsler L. Kemp S. (2005). Clinical importance of microbleeds in patients receiving IV thrombolysis. *Neurology* 65 1175–1178. 10.1212/01.wnl.0000180519.27680.0f 16247042

[B28] Larrue V. von Kummer R. R. Müller A. Bluhmki E. (2001). Risk factors for severe hemorrhagic transformation in ischemic stroke patients treated with recombinant tissue plasminogen activator: a secondary analysis of the European-Australasian Acute Stroke Study (ECASS II). *Stroke* 32 438–441. 10.1161/01.str.32.2.438 11157179

[B29] Lindsay M. P. Norrving B. Sacco R. L. Brainin M. Hacke W. Martins S. (2019). World Stroke Organization (WSO): global stroke fact sheet 2019. *Int. J. Stroke* 14 806–817. 10.1177/1747493019881353 31658892

[B30] Liu L. Luo H. Liu H. (2019). Influence of cerebral small vessel disease overall burden on clinical outcome of acute stroke patients after intravenous thrombolysis. *Chin. J. Cerebrovasc. Dis.* 16 393–399.

[B31] Liu X. Li T. Diao S. Cai X. Kong Y. Zhang L. (2019a). The global burden of cerebral small vessel disease related to neurological deficit severity and clinical outcomes of acute ischemic stroke after IV rt-PA treatment. *Neurol. Sci.* 40 1157–1166. 10.1007/s10072-019-03790-x 30830567

[B32] Liu X. Li T. Wang Z. (2019b). Relationship between total cerebral small vessel disease burden and hemorrhagic transformation of acute ischemic stroke patients after intravenous thrombolysis. *Chin. J. Neurol.* 52 209–215.

[B33] Liu X. Zhang J. Tian C. Wang J. (2020). The relationship of leukoaraiosis, haemorrhagic transformation and prognosis at 3 months after intravenous thrombolysis in elderly patients aged >= 60 years with acute cerebral infarction. *Neurol. Sci.* 41 3195–3200. 10.1007/s10072-020-04398-2 32358704 PMC7567704

[B34] Liu X. Y. Li T. Mei C. H. (2019). Recent advances in hemorrhage transformation and clinical prognoses after intravenous thrombolysis in acute ischemic stroke with cerebral small vessel disease. *Chin. J. Neuromed.* 18 481–486.

[B35] Liu Y. Zhang M. Chen Y. Gao P. Yun W. Zhou X. (2018). The degree of leukoaraiosis predicts clinical outcomes and prognosis in patients with middle cerebral artery occlusion after intravenous thrombolysis. *Brain Res.* 1681 28–33. 10.1016/j.brainres.2017.12.033 29288062

[B36] Liu Y. Zhang M. Yun W. Zhang Z. X. Zhou X. J. Yun W. W. (2017). Influence of moderate to severe leukoaraiosis on hemorrhagic transformation and prognosis of acute ischemic stroke patients after intravenous thrombolysis. *Chin. J. Neurol.* 50 885–891.

[B37] Matusevicius M. Paciaroni M. Caso V. Bottai M. Khurana D. de Bastos M. (2019). Outcome after intravenous thrombolysis in patients with acute lacunar stroke: an observational study based on SITS international registry and a meta-analysis. *Int. J. Stroke* 14 878–886. 10.1177/1747493019840947 30935349

[B38] Moriya Y. Takahashi W. Kijima C. Yutani S. Iijima E. Mizuma A. (2013). Predictors for hemorrhagic transformation with intravenous tissue plasminogen activator in acute ischemic stroke. *Tokai J. Exp. Clin. Med.* 38 24–27. 23564572

[B39] Nagaraja N. Tasneem N. Shaban A. Dandapat S. Ahmed U. Policeni B. (2018). Cerebral microbleeds are an independent predictor of hemorrhagic transformation following intravenous alteplase administration in acute ischemic stroke. *J. Stroke Cerebrovasc. Dis.* 27 1403–1411. 10.1016/j.jstrokecerebrovasdis.2017.12.044 29398533

[B40] No authors listed (1989). Stroke–1989. Recommendations on stroke prevention, diagnosis, and therapy. Report of the WHO task force on stroke and other cerebrovascular disorders. *Stroke* 20 1407–1431. 10.1161/01.str.20.10.1407 2799873

[B41] No authors listed (1997). Intracerebral hemorrhage after intravenous t-PA therapy for ischemic stroke. The NINDS t-PA Stroke Study Group. *Stroke* 28 2109–2118. 10.1161/01.str.28.11.2109 9368550

[B42] Pang J. Zhang J. H. Jiang Y. (2019). Delayed recanalization in acute ischemic stroke patients: late is better than never? *J. Cereb. Blood Flow Metab.* 39 2536–2538. 10.1177/0271678x19881449 31594437 PMC6893989

[B43] Pantoni L. (2010). Cerebral small vessel disease: from pathogenesis and clinical characteristics to therapeutic challenges. *Lancet Neurol.* 9 689–701. 10.1016/s1474-4422(10)70104-6 20610345

[B44] Park J. H. Ko Y. Kim W. J. Jang M. S. Yang M. H. Han M.-K. (2012). Is asymptomatic hemorrhagic transformation really innocuous? *Neurology* 78 421–426. 10.1212/wnl.0b013e318245d22c 22282643

[B45] Powers W. J. Rabinstein A. A. Ackerson T. Adeoye O. M. Bambakidis N. C. Becker K. (2019). Guidelines for the early management of patients with acute ischemic stroke: 2019 update to the 2018 guidelines for the early management of acute ischemic stroke: a guideline for healthcare professionals from the American Heart Association/American Stroke Association. *Stroke* 50 e344–e418. 31662037 10.1161/STR.0000000000000211

[B46] Rha J. H. Shrivastava V. P. Wang Y. Lee K. E. Ahmed N. Bluhmki E. (2014). Thrombolysis for acute ischaemic stroke with alteplase in an Asian population: results of the multicenter, multinational safe implementation of thrombolysis in Stroke-Non-European Union World (SITS-NEW). *Int. J. Stroke* 9(Suppl. A100) 93–101. 10.1111/j.1747-4949.2012.00895.x 22988894

[B47] Shi Y. Thrippleton M. J. Makin S. D. Marshall I. Geerlings M. I. de Craen A. J. M. (2016). Cerebral blood flow in small vessel disease: a systematic review and meta-analysis. *J. Cereb. Blood Flow Metab.* 36 1653–1667. 10.1177/0271678x16662891 27496552 PMC5076792

[B48] Staals J. Makin S. D. Doubal F. N. Dennis M. S. Wardlaw J. M. (2014). Stroke subtype, vascular risk factors, and total MRI brain small-vessel disease burden. *Neurology* 83 1228–1234. 10.1212/wnl.0000000000000837 25165388 PMC4180484

[B49] Staszewski J. Skrobowska E. Piusińska-Macoch R. Brodacki B. Stȩpień A. (2019). Cerebral and extracerebral vasoreactivity in patients with different clinical manifestations of cerebral small-vessel disease: data from the significance of hemodynamic and hemostatic factors in the course of different manifestations of cerebral small-vessel disease study. *J. Ultrasound Med.* 38 975–987. 10.1002/jum.14782 30208231

[B50] Terruso V. D’Amelio M. Di Benedetto N. Lupo I. Saia V. Famoso G. (2009). Frequency and determinants for hemorrhagic transformation of cerebral infarction. *Neuroepidemiology* 33 261–265. 10.1159/000229781 19641332

[B51] Tsivgoulis G. Zand R. Katsanos A. H. Turc G. Nolte C. H. Jung S. (2016). Risk of symptomatic intracerebral hemorrhage after intravenous thrombolysis in patients with acute ischemic stroke and high cerebral microbleed burden: a meta-analysis. *JAMA Neurol.* 73 675–683. 10.1001/jamaneurol.2016.0292 27088650

[B52] Turc G. Isabel C. Calvet D. (2014). Intravenous thrombolysis for acute ischemic stroke. *Diagn. Interv. Imaging* 95 1129–1133. 25465121 10.1016/j.diii.2014.10.002

[B53] van Leijsen E. Bergkamp M. I. van Uden I. Ghafoorian M. van der Holst H. M. Norris D. G. (2018). Progression of white matter hyperintensities preceded by heterogeneous decline of microstructural integrity. *Stroke* 49 1386–1393. 10.1161/strokeaha.118.020980 29724890

[B54] Wang H. Nie Z. Y. Liu M. Li R. R. Huang L. H. Lu Z. (2020). Clinical characteristics of perivascular space and brain CT perfusion in stroke-free patients with intracranial and extracranial atherosclerosis of different extents. *Ann. Transl. Med.* 8:215. 10.21037/atm.2020.01.35 32309362 PMC7154435

[B55] Wang S. Lv Y. Zheng X. Qiu J. Chen H. S. (2017). The impact of cerebral microbleeds on intracerebral hemorrhage and poor functional outcome of acute ischemic stroke patients treated with intravenous thrombolysis: a systematic review and meta-analysis. *J. Neurol.* 264 1309–1319. 27885484 10.1007/s00415-016-8339-1

[B56] Wardlaw J. M. Smith E. E. Biessels G. J. Cordonnier C. Fazekas F. Frayne R. (2013). Neuroimaging standards for research into small vessel disease and its contribution to ageing and neurodegeneration. *Lancet Neurol.* 12 822–838. 23867200 10.1016/S1474-4422(13)70124-8 PMC3714437

[B57] Wells G. A. Shea B. Higgins J. P. Sterne J. Tugwell P. Reeves B. C. (2013). Checklists of methodological issues for review authors to consider when including non-randomized studies in systematic reviews. *Res. Synth. Methods* 4 63–77. 10.1002/jrsm.1077 26053540

[B58] Wen L. Guofang C. Yixin L. Weitvei L. Lei P. Shengku Z. (2019). White matter hyperintensities and prognosis of patients with acute cerebral infarction treated by intravenous thrombolysis with alteplasejan analysis of influencing factors. *Chin. J. Cerebrovasc. Dis.* 16 508–513.

[B59] Xue J. Wang H. Gao P. Y. (2017). Study on correlation between cerebral microbleeds and hemorrhagic transformation after thrombolytic therapy in acute ischemic stroke. *Chin. J. Stroke* 12 477–483.

[B60] Yan S. Jin X. Zhang X. Zhang S. Liebeskind D. S. Lou M. (2015a). Extensive cerebral microbleeds predict parenchymal haemorrhage and poor outcome after intravenous thrombolysis. *J. Neurol. Neurosurg. Psychiatry* 86 1267–1272. 10.1136/jnnp-2014-309857 25632155

[B61] Yan S. Mao Y. Zhong G. Zhang S. Lou M. (2015b). Safety of intravenous thrombolysis in cerebral microbleeds patients with prior antiplatelet therapy. *Zhejiang Da Xue Xue Bao Yi Xue Ban* 44 618–624. 26822043 10.3785/j.issn.1008-9292.2015.11.04 PMC10397054

[B62] Yan S. Q. Wan J. P. Guo Y. Zhang S. Lou M. (2014). [Impact of cerebral microbleeds on outcomes of acute ischemic stroke treated with intravenous thrombolysis]. *Zhejiang Da Xue Xue Bao Yi Xue Ban* 43 20–27. 24616457 10.3785/j.issn.1008-9292.2014.01.019

[B63] Zhang D. P. Yin S. Zhang H. L. Li D. Song B. Liang J. X. (2020). Association between intracranial arterial dolichoectasia and cerebral small vessel disease and its underlying mechanisms. *J. Stroke* 22 173–184. 10.5853/jos.2019.02985 32635683 PMC7341005

[B64] Zhang Y. X. Zhang P. L. (2018). Safety of intravenous thrombolytic therapy with alteplase in patients with acute cerebral ischemic stroke complicated with cerebral microbleeds. *J. Apoplexy Nerv. Dis.* 34 314–316.

[B65] Zheng T. H. Hao J. J. Zhou X. Y. (2012). Analysis of related factors of early intracranial hemorrhage after intravenous rt-PA thrombolysis in advance-aged patients with cerebral infarction. *Chin. J. Cerebrovasc. Dis.* 9:362.

[B66] Zhuo Z. L. Nie Z. Y. Liu Y. H. (2020). Correlation between total burden of cerebral small vessel disease and outcome after intravenous thrombolysis in acute ischemic stroke patients. *Chin. J. Stroke* 15 734–739.

