# Supplementary material for: Neuroimaging Markers of Cerebral Small Vessel Disease on Hemorrhagic Transformation and Functional Outcome After Intravenous Thrombolysis in Patients With Acute Ischemic Stroke: A Systematic Review and Meta-Analysis
Source: Front Aging Neurosci. 2021 Jul 13;13:692942. doi: 10.3389/fnagi.2021.692942 (PMC8315270; doi:10.3389/fnagi.2021.692942)
Supplement: Supplementary file 1 [file Data_Sheet_1.PDF]

Supplementary

Appendix 1

Select Resource(s) to search:

Search Laurentian University' s Ovid Books
Search Ovid Table of Contents and Abstracts
Search Laurentian University' s Ovid Full Text Journals
EBM Reviews-Cochrane Central Register of Controlled Trials
EBM Reviews-Cochrane Database of Systematic Reviews
EBM Reviews-Cochrane Clinical Answers
EBM Reviews-Cochrane Methodology Register
EBM Reviews Full Text- Cochrane DSR, ACP Journal Club, CCA, and DARE
All EBM Reviews- Cochrane DSR, ACP Journal Club, DARE, CCA, CCTR, CMR, HTA, and NHSEED
AMED (Allied and Complementary Medicine)
Embase
Ovid Healthstar
Ovid MEDLINE(R) and Epub Ahead of Print, In-Process & Other Non-Indexed Citations, Daily and Versions(R)
Ovid MEDLINE(R) and Epub Ahead of Print, In-Process & Other Non-Indexed Citations and Daily
All Ovid MEDLINE(R)
Ovid MEDLINE(R) and Epub Ahead of Print, In-Process & Other Non-Indexed Citations and Daily
Ovid MEDLINE(R) and Epub Ahead of Print, In-Process & Other Non-Indexed Citations and Daily-without Revisions
Ovid MEDLINE(R) Epub Ahead of Print
Ovid MEDLINE(R) In-Process & Other Non-Indexed Citations
Ovid MEDLINE(R) Daily Update
Ovid MEDLINE(R)

Search history

1. (Ischemic stroke or cerebral infarction or Cerebral ischemia or ischemic attack or Cerebral ischemia or Cerebral infarction).mp.[mp=ti, ot, ab, sh, hw, kw, tx, ct, tn, dm, mf, dv, fx, dq, nm, kf, ox, px, rx, an, ui, sy]
2. (post-thromboly* or thromboly* or tPA or rtPA or rt-PA or (tissue plasminogen activator) or thrombectomy).mp.[mp=ti, ot, ab, sh, hw, kw, tx, ct, tn, dm, mf, dv, fx, dq, nm, kf, ox, px, rx, an, ui, sy]
3. (cerebral small vessel disease or leukoaraiosis or white matter hyperintens* or white matter lesion* or white matter disease* or white matter change* or microbleed* or "small vessel":or microbleed* or infarct* or enlarged perivascular space or EPVS).mp.[mp=ti, ot, ab, sh, hw, kw, tx, ct, tn, dm, mf, dv, fx, dq, nm, kf, ox, px, rx, an, ui, sy]
4. 1 and 2 and 3

Appendix 2

The details of quality assessment

Author(year)	Selection				Comparability	Outcome			Total
	Representativeness of the exposed cohort	Selection of the non esposed cohort	Ascertainment of the exposure	Demonstration that outcome of interest was not present at start of study	comparability of cohorts on the basis of the design or analysis	Assement of outcome	Was follow-up long enough for outcomes to occur	Adequacy of follow up of cohorts	
Moriya Y 2013	1	1	1	1	1	1	0	0	6
Capuana,M.L 2020	1	1	1	1	1	1	1	1	8
Liu,X 2019	1	1	1	1	1	1	1	1	8
Wen,L 2019	1	1	1	1	1	1	1	1	8
Liu,L 2019	1	1	1	1	1	1	1	1	8
Liu,X.Y 2019	1	1	1	1	1	1	0	0	6
Chacon-Portillo 2018	1	1	1	1	1	1	0	0	6
Nagaraja,N 2018	1	1	1	1	1	1	0	0	6
Liu,Y 2018	1	1	1	1	1	1	1	1	8
Liu,Y 2017	1	1	1	1	1	1	1	1	8
Yan,S.Q 2015	1	1	1	1	1	1	1	0	7
Curtze,S 2015	1	1	1	1	1	1	0	0	6
Dannenberg,S 2014	1	1	1	1	1	1	1	1	8
Yan,S.Q 2014	1	1	1	1	1	1	1	1	8
Zheng,T.H 2012	1	1	1	1	1	1	1	0	7
Kakuda,W 2005	1	1	1	1	1	1	1	1	8

Derex,L 2004	1	1	1	1	1	1	0	0	6
Yan,S 2015	1	1	1	1	1	1	0	0	6
Dong,J 2018	1	1	1	1	1	1	0	0	6
Zhang,Y 2018	1	1	1	1	1	1	0	0	6
Huang,S 2017	1	1	1	1	1	1	0	0	6
Zhuo,Z 2020	1	1	1	1	1	1	1	1	8
Xue,J 2017	1	1	1	1	1	1	0	0	6
Curtze, S 2015	1	1	1	1	1	1	0	0	6
